# Patients’ experiences of quality in palliative care and advance care planning in primary care: a qualitative study

**DOI:** 10.1186/s12904-025-01919-8

**Published:** 2025-11-03

**Authors:** Liv Skomakerstuen Ødbehr, Aasa Serholt Jensen, Aina Korup, Reidun Hov, Harald Sanaker, Tuva Sandsdalen

**Affiliations:** 1 Department of Health and Nursing Sciences, University of Inland Norway, Faculty of Social and Health Sciences, Post Box 400, Elverum, 2418 Norway; 2 Centre for Development of Institutional and Home Care Services, Hamar municipality, Norway; 3 Municipal Medical Centre Ringsaker municipality, Brumunddal, Norway

**Keywords:** Advance care planning, Quality of care, Palliative care, Patients’ experiences, Primary healthcare

## Abstract

**Background:**

Primary healthcare services provide care to patients and their relatives during the late palliative phase in the patients’ home or in facilities such as nursing homes. Patients receiving palliative care may be vulnerable due to the total pain of the situation they are facing, which requires great expertise of healthcare professionals in meeting the patients’ needs. The aim in this study was to explore patients’ experiences of quality in palliative care and advance care planning in primary healthcare services.

**Methods:**

This study has a qualitative design. Five patients in the late palliative phase in primary care from different municipalities in Norway participated. All participants had experiences across both primary and specialist care settings. Individual interviews were conducted between January 2022 and July 2023. The interviews were transcribed verbatim and analysed using content analysis. The study adheres to the checklist of the consolidated criteria for reporting qualitative research.

**Results:**

The findings are described in terms of four main themes: (1) trust in supportive clinicians to ease their future path, (2) perceived quality of palliative care depended on the context, (3) advance care planning helped to tidy up important aspects of life, and (4) personalized family support and involvement eased burdens for patients and relatives.

**Conclusion:**

Patients in late palliative phase and their experiences of palliative care highlight the need for information and support from healthcare professionals, but the organization of primary healthcare settings does not adequately address these needs. A key aspect of quality in care is comprehensive and individualized follow-up throughout the patient’s illness trajectory. Another area that affects the quality of palliative care is the communication skills of healthcare professionals. Care plans for the end- of -life were developed through advance care planning, but the quality of implementation varied in home care. A huge concern for patients in the late palliative phase was if and how their relatives received the support they needed.

**Supplementary Information:**

The online version contains supplementary material available at 10.1186/s12904-025-01919-8.

## Background

Palliative care aims to improve quality of life in severe life-threatening illness; pain and symptom relief are key elements, irrespective of the patient’s diagnosis [1]. Patients in the late palliative phase may receive primary palliative care from nurses in their home or in facilities such as nursing homes, due to that late palliative phase is characterized by patients have a life-threatening illness expected to progress towards death within one year [2, 3]. Palliative care in this phase comprises symptom relief for the physical, psychosocial and existential suffering of patients as well as care for the patients’ relatives in line with principles of holistic care [1, 4–6]. Further, palliative care implies continuous care during the entire illness trajectory and comprises a person-centred approach that involves responding to patients’ individual needs and preferences [7]. Patients receiving palliative care may be vulnerable due to the total pain of the situation they are facing. Palliative care therefor requires expertise to ensure that the patients’ needs are met [4]. A recognized way of addressing patients’ needs and values is advance care planning [8]. Here, nurses play an important role in both planning and implementing palliative care.

According to the European Association of Palliative Care, advance care planning means that individuals may define their wishes and goals for future medical treatment and care, as well as their preferences for the end- of -life, in collaboration with their family members and healthcare professionals [8]. Advance care planning is a process of ongoing communication where the patient can record, and review decisions and preferences as needed [8, 9]. It involves clarifying expectations and reinforcing hope and empowerment in patients’ lives. This has been referred to as a medical art [10]. Furthermore, there are instruments to help healthcare professionals identify people with deteriorating health due to life-limiting conditions, including frailty in older age. An example is the Supportive and Palliative Care Indicators Tool [11]. Advance care planning also involves an exploratory process of the patient’s prognostic awareness, aiming to offer the patient advice if needed and strengthen the patient’s experience of coping [12, 13]. The patient is invited to a dialogue with healthcare professionals about what is important in life here and now and at the end- of- life [9, 14, 15]. Such conversations are vital for care and treatment planning and provide predictability and reassurance, being based on the patient’s wishes and needs [8, 16, 17]. The aims of such dialogues are to enhance patients’ quality of life and dignity, as well as their right to self-determination and participation in palliative treatment and care [16]. Although the European Association’s definition of advance care planning is acknowledged internationally, we find varied experiences of advance care planning in Norwegian municipal (primary) care [7]. National guidelines recommend advance care planning if the patient has a short life expectancy, while experience suggests that dialogue and planning should start at an early stage of the disease [7, 18]. The Norwegian authorities have developed guidelines for a systematic introduction of advance care planning in primary care [7, 18]. The aim is to offer patients medical care, advice, and guidance. However, the heavy workload of healthcare professionals and poor collaboration often hamper the implementation of advance care planning [7, 16, 19]. Some research has been conducted on advance care planning in hospitals and nursing homes [20–24], but relatively few studies deal with patients’ experiences of advance care planning in primary healthcare [21].

Quality of care is commonly defined that health services should be effective, safe, person-centred, timely, equitable, integrated and efficient [25]. To provide quality of palliative care, it is essential to implement the principles of palliative care as describes above [1]. ACP is in this regard a tool to ensure that care is in line with these principles [1, 8, 9].

Quality of care is multidimensional and may differ depending on the perspective [26]. Patients’ perceptions of what constitutes quality in primary care is important to improve the services [4]. They are shaped and defined by norms, expectations and experiences from previous encounters with healthcare professionals [17, 26]. Patients’ views are sometimes related to subjective and personal factors such as age, gender, level of education and physical and mental well-being [20]. Other key factors might be external and objective aspects such as relieving of pain and other symptoms in the patients’ lives, competence and skills of nurses and other healthcare professionals, physical environment, cooperation between different healthcare services, provision of proper equipment, as well as nurse-patient communication including respect for patients’ autonomy and person-centredness [8, 20, 26, 27]. Patients living at home often receive a complex range of support in primary healthcare, including short-term stays in a healthcare facility [1, 21, 23]. Due to limited resources leading to poor continuity and interaction between healthcare professionals in primary healthcare services, patient care is often perceived to be task-driven and fragmented, in contrast to person-centred care [19]. This is in line with a scoping review who identified challenges in delivering nursing care for patients who preferred to die at home [27]. The review revealed several barriers for enabling home-death related to lack of resources in forms of limited availability of healthcare professionals as well as a lack of equipment and nursing supplies. The review also emphasises the need for palliative care based on knowledge and competence which made patients feel safe. Research also shows that patients receiving palliative care emphasize the safeguarding of values like comfort, trust, hope and respect [10]. Other studies uncovers that patients want to participate in everyday nursing care decisions related to the end -of -life by an open and honest communication with healthcare professionals [23, 28–30]. This might be in line with the findings in a qualitative study of Nysæter et al. [31] who showed that patients’ and their relatives in home care wanted palliative care to be organised in a way that ensured continuity and predictability especially related to communication and care offerings, as well as advise on further treatment and care. A systematic review from 2021 [32], concluded with that patients receiving end-of‐life home‐based care reports greater satisfaction of care delivered than those who die in hospitals. Nevertheless, the review highlights that despite the widespread wish to support patients at the end- of -life, there are limited evidence supporting the effectiveness of home‐based end‐of‐life care even though there are some as mentioned. This reveals that there is a need for more research to explore patients’ experience and perceptions of palliative care and advance care planning in the primary healthcare context.

## Methods

The aim of this study was thus to explore patients’ experiences of quality in palliative care and advance care planning in primary healthcare services. By asking patients themselves, we can access their experiences of these important aspects of care. The research question was as follows: How do patients experience the quality of palliative care and advance care planning in primary healthcare services?

### Design

This study has a qualitative design, based on individual interviews with patients in the late palliative phase. A qualitative design was used as it was best suited to answer the research question and to provide an understanding of the topic under study. Content analysis [33] was used to elicit both manifest and latent content from the interviews, with an inductive approach to the analysis of patient experiences. The study adheres to the Consolidated Criteria for Reporting Qualitative Research checklist [34] (Supplementary material 1).

### Study setting and recruitment

Primary healthcare services in Norway provides care for people in their own homes. Primary healthcare also encompasses institutional care, personal and practical assistance as well as support for patients’ relatives [24]. This study was conducted in the primary care setting in different municipalities in Norway. In this context, patients receive primary care from healthcare professionals with general or specialized expertise in palliative care [7]. Primary care services provide care to patients and their relatives during the late palliative phase in the patients’ home or in facilities such as nursing homes. In the home care context, nurses and general practitioners cooperate with other healthcare professionals as needed. Some local authorities have established palliative care teams around patients. The palliative care team has a consultative function and is sometimes supplemented with a cancer coordinator to assist patients and their relatives. Most palliative patients need short-term or long-term stays in a primary or secondary healthcare facility because of their increasing needs for care. The aim is for example, to provide patients with relaxation, optimize symptom relief and create a sense of security. Participants in this study were recruited from home care and nursing homes.

### Inclusion and exclusion criteria

Participants were included in the study based on a purposive sampling method. The inclusion criteria were (i) patients who had received a written invitation to an advance care planning meeting in the previous two weeks, (ii) those who remembered the content of the meeting, (iii) those who could participate in an interview of 30–40 min, and (iv) all participants needed to be cognitively healthy and able to give consent. Exclusion criteria were patients who were not in the late palliative phase or otherwise did not meet the inclusion criteria.

A specialist nursing home nurse and a cancer nurse coordinator recruited potential participants on behalf of the research group. Requests were first sent to primary care leaders in several local authorities in three large counties in southern Norway. Two members of the research group subsequently sent further requests for recruitment of patients to leaders of home care, nursing homes, short-term wards and health centres. It was challenging to recruit patients to the study, since many healthcare professionals expressed that these patients were too ill to participate in an interview. However, five patients agreed to participate. Three of them were recruited from short-term wards with palliative care beds to recover for a few days and two were receiving home care when the interview was conducted.

### Characteristics of the participants

The group of participants consisted of one man and four women. All participants had been diagnosed with cancer and most had different chronic comorbidities, undergoing treatment in the late palliative phase of their illness. All participants had experiences across both primary and specialist care settings. The patients’ age ranged from 51 to 85 years. No participants who were invited declined or dropped out from the study. All five spontaneously and independently expressed their appreciation at being part of the study to enable their experience to benefit others in the future.

The advance care planning was conducted by doctors and/or a nurse (cancer coordinator) and the patient’s family members were also invited to take part in the advance care planning meeting. The nursing home ward had guidelines for advance care planning based on advice from the “Centres for Medical Ethics” [35]. The content and conclusions of the advance care planning meeting were documented in a separate form in the patient record.

### Data collection

The period of data collection was from January 2022 to July 2023. Two female registered nurses from the research group, both working in primary healthcare, conducted the interviews. One of them worked in a nursing home with a palliative care unit and the other was involved in primary healthcare quality improvement through professional and service development. A semi-structured interview guide was developed by the research group to cover the aim of the study (Table 1). To ensure that the interview guide was easy to understand and relevant to the study aim, the interview questions were first discussed in the research group and then piloted in the interview with one of the participants. The piloting did not lead to any changes in the guide, and the pilot interview was thus included in the study. The participants in the study did not know the researchers in advance. No one else was present during the interviews besides the researcher and the patient. One interview was conducted with each patient. Notes were taken during the interviews to facilitate follow-up questions. The interviews were audio-recorded and transcribed immediately afterwards. None of the patients read the transcripts after the interview because their condition rapidly deteriorated.

**Table 1:** Examples of questions from the interview guide

Q 1	What was your experience of the palliative care you were offered?
Q 2	How did you experience to participate in advance care planning?
Q 3	How did you feel about the follow-up?

### Data analysis

The text was analysed using content analysis as described by Graneheim and Lundman [33, 36]. Each recorded interview was listened to by all members of the research group. The interviews were transcribed verbatim and simultaneously read and checked for accuracy. First, each author was asked to carefully read the interview transcripts and suggest meaning units which corresponded with the aim of the study. Then, all authors in the project group discussed and identified the meaning units together. The meaning units were condensed, abstracted and labelled with codes. All members of the research group coded data. The codes were then compared based on similarities and differences, and similar codes were grouped together with a new designation in a sub-theme. The identification of the sub-themes was an iterative process between the steps of the analysis, and the sub-themes were discussed by the authors until consensus was reached. The sub-themes consisted of the manifest content of the analysis. Finally, the sub-themes were discussed in view of the latent content, which led to the formulation of themes. This constituted the inductive part of the analysis. Sub-themes and themes are presented in Table 2.

**Table 2:** Sub-themes and themes

Sub-themes	Themes
The need for guidance and advice on an unclear future path	Trust in supportive clinicians to ease their future path
The need for predictability and follow-up care from healthcare professionals	
The need for a sense of belonging	Perceived quality of palliative care depended on the context
Receiving care from someone they were familiar with	
“Tidying up” at the end of life	Advance care planning helped to tidy up important aspects of life
Dealing with an emotional crisis	
The patient’s family must be involved	Personalized family support and involvement eased burdens for patients and relatives
The family was involved in care	

### Rigour and reflexivity

The research group, consisting of five nurses and one doctor, had a combination of clinical competence and research experience from palliative care in various contexts including nursing homes. The researchers were aware of that personal experiences and knowledge in the field of palliative care could affect the questions they asked the participants during the interviews. Each of the group-members acknowledged that prior experiences, assumptions and beliefs could influence the research process and therefor all group members participated in the analysis- process by joint discussions and reflections. Being aware of one’s own background experience, preconception and prejudices was seen as a way of avoiding researcher bias [36].

## Results

The findings from the content analysis of patients’ experiences of quality in palliative care and advance care planning in primary healthcare services based on the interviews are presented under the following themes: (3.1) Trust in supportive clinicians to ease their future path, (3.2) Perceived quality of palliative care depended on the context, (3.3) Advance care planning helped to tidy up important aspects of life, and (3.4) Personalized family support and involvement eased burdens for patients and relatives.

### Trust in supportive clinicians to ease their future path

The participants found that the healthcare professionals were supportive and strongly rooted in their professional values, and that they tried to create a safe framework and predictability in personalized care. Several of the participants stated that they trusted the health care professionals to make sound and correct decisions concerning the treatment offered. The reassurance that someone was taking care of the overall aspects of their situation was of utmost importance in providing a sense of security and relief and this was associated with good quality care.

This trust in healthcare professionals was important during the whole process as the participants dealt with their illness individually and variously from time to time and did not always understand what the best possible care was for them. They often acknowledged retrospectively their needs for guidance and advice from the healthcare professionals and realized, often too late, that they had declined the assistance they were offered. One of them expressed this as follows: “*In my head*,* and with my background*,* the term ‘palliative team’*,* is the same as terminal. And then I didn’t know what was best for me*” (Participant 1).

The participants were trying to sort out their thoughts and feelings together with the health care professionals. Particularly in one-to-one conversations with the healthcare professionals they received support to express their needs and preferences and gain deeper insight into their situation. Such dialogues were highly appreciated by the participants, and some expressed the need for even closer dialogues, especially shortly after what they felt was “bad news”. Receiving care, understanding and respect from healthcare professionals was felt to ease their situation:

“*That’s what was so strange*,* I can say with my hand on my heart that I haven’t shed a tear over the fact that I’ve been diagnosed with cancer*,* but sometimes a tear comes when I think about how many good people I have around me*,* so then I feel so grateful*.” (Participant 4).

They also appreciated the fact that the content of the conversation could be passed on to other healthcare professionals; they felt that this relieved them from troublesome thoughts. One said: “*What I wanted to say … and what I was concerned about … was then written down and that’s fine. Now I don’t have to think about it any longer*,* it’s gone.*” (Participant 3).

### Perceived quality of palliative care depended on the context

Short-term stays were important to the four participants who had taken up the offer. Firstly, they stated that the short-term units gave them a safe and comfortable atmosphere that was suitable for their situation. Secondly, they appreciated the short time between expressing a need and having the need met, especially when they had experienced loss of functioning over time. The close contact with healthcare professionals reassured them that care would be provided just when they needed it and they did not have to wait long for answers to their questions. This was valuable since they felt more tired as the disease progressed, which made their condition change quite rapidly. In this phase of the disease, they needed continuity of care from healthcare professionals. One of the participants said: “*It’s as if they’ve got eyes on each finger …even if I don’t ask for help*,* they’re mind readers.*” (Participant 4) “*The care I receive is one hundred and ten%. I get good information I can understand*,* and tailored symptom relief.*” (Participant 4).

For the participants, certain basic things were important, such as being seen and treated as a human being: “*The fact that you’re not just seen as your illness. You’re a human being with that disease. Things are not taboo*” (Participant 5). Another valuable benefit was the contact they established with other patients in short-term wards. A feeling of community between people in the same situation led to acknowledgement, identification with each other and the development of deep friendships.

The participants felt insecure at returning home from short-term wards because they found home care to be different and poorly coordinated at times. Different healthcare professionals were perceived as having separate fields and tasks and the participants missed better cooperation between health care professionals in primary care.

In the participants’ homes, they experienced a less close relationship with healthcare professionals. They sometimes felt left to themselves at home with insufficient support. This was particularly evident immediately after they had realized the gravity of the diagnosis. In such situations they expressed feelings of vulnerability, loneliness and deep insecurity. They described how they were left alone to deal with the emotional crisis immediately after being diagnosed, needing predictability and care in their emotional rollercoaster. One of the participants said that no one asked about her for a fortnight after the diagnosis was established. Follow-up care was perceived as distant, and she lacked a contact person:

“*There are many people to relate to*,* and I miss someone who’d stick with me. I felt like I was hanging in mid-air*,* I had to make contact myself*,* and I missed someone getting in touch with me.*” (Participant 1).

The participants pointed out the need for closer monitoring in the early phase of the disease, not only in the late palliative phase, which they found positive. They described how care needed to be individualized, and they especially appreciated receiving this from doctors and the way doctors cared about their whole situation. Care needs increased as the disease progressed.

### Advance care planning helped to tidy up important aspects of life

Advance care planning was seen as part of palliative care in the healthcare service to uphold the quality in palliative care, thus not a separate issue. Advance care planning helped to ensure that the information and care provided by healthcare professionals was tailored to each of them individually. One description was: “Advance care planning *was an open*,* honest and pleasant conversation*,* considering what it was about*” (Participant 5). The participants stated that advance care planning involved different obligations and greater responsibility. One advantage of advance care planning was that it is a planned meeting, unlike a spontaneous bedside conversation. advance care planning helped them to reflect on their situation and enabled them to speak about what was on their minds. The participants saw it as a form of “tidying up” mentally and practically. One participant said: “Advance care planning *is a way to get rid of the difficult and painful things but instead keep the important aspects of life*” (Participant 3). They also said that in some matters they changed their way of thinking as the disease progressed; this applied to wishes and needs that they did not realize were important until a later stage of the disease.

However, advance care planning itself initiated a process where subsequent reflections emerged. Such reflections needed to be further addressed and discussed. The participants therefore found it important to have a system where healthcare professionals could follow up on their needs for support after an advance care planning. One participant said:

“*Because there’s no point if you’re invited to an* advance care planning *and then after six months*,* you’re offered a place in a nursing home and then you die soon after that. There would have been no point in arranging an* advance care planning *meeting if there wasn’t any contact in the six months in between.”* (Participant 5).

For the participants, advance care planning was valuable in helping them to articulate their wishes for the rest of their life, which also made them appreciate life, take good care of the time remaining and have a sense of living well as long as possible. ACP helped them to articulate their wishes so that the quality of care could be maintained through forward planning. One participant expressed it in this way:

“*You know*,* it just felt good afterwards. I was embarrassed*,* but afterwards it was just good to talk about it. Because it’s not the kind of thing you talk much about… death and things like that. So*,* I think it did something good to me.*.” (Participant 2).

Advance care planning was described as an eye-opener in relation to feelings that had not been put into words. Sometimes advance care planning meetings were harrowing and difficult, but over the longer term the participants felt that advance care planning gave them a sense of relief and had a positive influence on them by reassuring them that the quality of care during the remaining time would be good.

Some participants expressed a mixed view of their situation. On the one hand they had hoped for a cure, while on the other hand they gained an understanding of the seriousness of the fact that they would die of the disease. One of them said:

“*Yes*,* I have to try to get back on my feet. And then I’d like to start working again. That’s really my goal. Because I think that gives me so much. Getting up in the morning*,* meeting colleagues. As long as I’m alone*,* then it’s very good to get out*,* that’s important. I may not be able to do it*,* but I hope so…. What I hope is that I’ll come back. But if I can’t do it*,* well*,* I can’t do it. We’ll have to take things as they come. I’m supposed to live my life*,* not just at work. And as long as possible…*” (Participant 3).

Some of the participants also described how the level of their hopes changed in line with the development of the disease and increased openness about what was to come.

### Personalized family support and involvement eased burdens for patients and relatives

A sense of community and belonging was considered important and beneficial, and the participants saw their family as a resource which they wanted to have close to them. They felt that they and their family were in the “same boat*”*. To involve their family members in advance care planning and decisions regarding the disease and treatment was reassuring and increased the perception of that the quality of palliative care was good. One participant put it this way: “*It’s nice to have an overall view of your loved ones. They help us to be less lonely together - in these difficult days*” (Participant 3). To talk about the future death in the advance care planning with their family gave them a kind of courage:

“*Basically*,* I’ve been open about my situation all the time*,* for better or worse. And I think that’s helped me a lot now. I find it difficult to tell the truth to my loved ones or answer questions and so on*,* so I think that helps me - feeling brave. Brave enough to express what I feel. Because people aren’t mind readers.*” (Participant 4).

The participants valued openness and honesty as far as possible, which included their will after death. Openness helped them to work through the situation and prepare for death. However, not all participants found it easy to talk to their family about the disease. Sometimes this also applied to their spouse. One said: “*My husband is a bit more vulnerable*,* so he gets information afterwards*,* and then I think he handles it better*” (Participant 2).

The participants also reflected on the importance of talking about practical matters related to their funeral and the time after the funeral, stating: “*My relatives can then spread the work and responsibilities between them for looking after the practicalities after my death*” (Participant 3). Such conversations were also part of the grieving process and a way to find closure.

Useful information from healthcare professionals to the family also helped to ease the burdens for the patients. The participants were concerned about the needs of their family members, saying that they also needed care in the form of support and advice to handle the situation.

## Discussion

The aim in this study was to explore patients’ experiences of quality in palliative care and advance care planning in primary healthcare services. The findings show that patients in the late palliative phase emphasize the need for support from healthcare professionals and comprehensive care where all aspects of their lives are addressed. The patients’ situation influenced the kind of care they were offered and patients valued continuity of care involving good end-of-life care plans. Advance care planning was appreciated as a communication process when it came at the right time in the patients’ illness trajectory. Furthermore, the patients had a strong desire for their relatives to receive care. The findings are discussed below.

The need for a person-centred and holistic approach to care was underlined by the participants in the present study in line with WHO [25] definition of quality in care. They appreciated the fact that healthcare professionals talked about choices beyond symptom relief to address their needs in the here and now and in the future. They felt that healthcare professionals could support them in reflections on the end of life and be of great help in processing difficult questions. Recent studies also confirm this by emphasizing the need for healthcare professionals to talk openly with patients about all relevant issues, including existential matters [10, 15]. Basic individual needs such as patients’ self-determination, identity and positive self-perception, which are particularly important at the end of life, must be acknowledged by healthcare professionals [4]. A literature review by Zwakman et al. [12] found that patients wanted to be open about their future preferences for help and care but found it difficult to express them. However, communicating their difficulties to healthcare professionals reassured and satisfied them. A study by Viftrup et al. [37] showed that many patients try to preserve their dignity and quality of life when experiencing major losses in life, as seen in end-of-life care. The healthcare professionals in that study expressed that including patients in different kinds of decisions provided a sense of belonging that meant a great deal to the patients. This is also consistent with the findings of the present study. The participants felt a closer connection to healthcare professionals who were not afraid to give them honest answers even though this was sometimes almost unbearable.

The findings of this study indicate the need for continuity and close individual follow-up care by a familiar healthcare professionals during the whole illness trajectory in order for patients to feel that the palliative care was of high quality. A review that investigated patients’ preferences for palliative care in various contexts found that patients wanted the care to be organized in line with their preferences, i.e. help when needed and continuity of care, particularly if provided by the same nurses and doctors that they knew personally [38].

However, the findings of the present study showed variation in patients’ views on the quality of palliative care in the two care contexts. Patients in home care found a lack of continuity [39]. A previous review has confirmed that patients’ needs are unmet in palliative home care [40]. Further, perceptions of care quality might depend on the people involved, organizational factors and contexts, with the least favourable experiences of palliative care quality found in the home care context [26, 41]. A recently completed project named “The Home Time and Home Death Development Program (2021–2024), initiated by the Norwegian Directorate of Health focused on palliative care in the home care context [42]. Several municipalities participated in the development program and designed a model for palliative care adapted to the municipality’s own organization. The intention was to facilitate home death for patients with serious and life-threatening illness. Through the project, several development areas were recognized including care for the patient, common routines, interdisciplinary collaboration, involvement of relatives, training programs on palliative care for healthcare professionals, and increasing awareness among patients about the possibility of dying at home. Further research should focus on how to improve palliative care in the home care setting to meet the individual needs of patients.

Advance care planning was experienced as an integral part of palliative care and the quality of the care services provided. Structured and planned advance care planning initiated a process of reflection, which could be challenging to deal with, but also had therapeutic value by helping patients to articulate their care preferences and enable “tidying up” and acknowledging what the important aspects of life were. However, the need for further advance care planning conversations and an individual care plan was strongly emphasized as important for the patients to view advance care planning as relevant and meaningful. These findings concur with the aim of conducting advance care planning, namely that advance care planning is a process comprising several meetings to discuss patients’ preferences for care and a palliative care plan based on these preferences [8, 9, 43]. One could argue that conducting an advance care planning with patients, both ethically and according to principles of palliative care, requires the decisions made to be implemented. The findings show that inviting patients to talk about their preferences for care and death leads to reflections on their present situation and existential issues, which could be difficult to handle for patients alone. It is therefore important for healthcare professionals to fulfil their ethical and professional obligation to follow up on decisions after performing advance care planning. Unfortunately, previous studies from nursing homes have found that healthcare professionals reflected little about the purpose and content of advance care planning, and how and when it should be conducted [16], and that lack of time and competence are barriers to providing advance care planning [44]. A whole-ward approach with a clearly defined impact on nursing home routines and engagement of staff, patients and their next of kin have been shown to facilitate successful implementation of advance care planning [21]. Further studies should focus on how to best implement advance care planning, including meetings in the home care context.

The participants in the present study were concerned about their immediate family and the care their family members received throughout the illness. Studies from nursing homes show that relatives are sometimes involved in care decisions [30], but that the patient’s voice is silenced, preventing patients from discussing their needs [19]. In home care, experiences varied in the present study. Several participants wanted their family to be involved throughout their illness trajectory. However, it was challenging and sometimes daunting to give information to their spouse and children. In a study of interviews with doctors in home care about “end-of-life breakpoint communication”, it was found that although the doctors were keen to inform patients and relatives, they had inadequate training in communication about difficult topics [45]. The participants in the present study found that healthcare professionals were generally more comfortable talking about death and the end of life in nursing homes. Patients living at home had to initiate such conversations themselves with their children and spouses. The close contact with healthcare professionals s in nursing homes provided opportunities to talk, while patients living at home missed regular conversations. This study indicates a need for competence building of healthcare professionals in home care to enable them to provide holistic care in a demanding end-of-life process to patients greatly weakened by their condition.

### Strengths and limitations of the study

Lincoln and Guba [46] used four pillars to assess trustworthiness in qualitative research and refer to credibility, transferability, dependability and conformability. This study included patients in the late palliative phase with experience of advance care planning. Few previous studies have investigated this perspective. The participants had different cancer diagnoses and varied in age and care setting. Including patients with different characteristics may provide variation and diversity in the data, which is considered a strength in qualitative content analysis [33, 36].

The trustworthiness of the findings may be influenced by the researchers’ preunderstanding and thereby compromise the credibility. In the analysis of the data, credibility was ensured through continuous dialogue between the researchers until consensus on the content and labelling of the themes was reached. This was to ensure that the themes were congruent with the content of the interviews.

Transferability refers to how well results can be transferred to other settings [33]. The patient characteristics and the care context of this study have been described to enable the reader to assess the transferability of the findings. The healthcare settings in which the participants were recruited were in the initial phase of implementing advance care planning, which may have influenced the recruitment process and the findings of this study. The patients in this study were introduced to advance care planning late in their illness trajectory and were in a late palliative phase when interviewed. The results might have been different for patients referred to advance care planning at earlier stages of their illness and the findings must therefore be transferred to those groups of patients with caution. Another aspect is that all participants were cancer patients. Other diagnostic groups with palliative care needs may have even less access to appropriate services.

Dependability refers to the stability of findings over time [46]. The interview guide was piloted to ensure that the questions were in line with the aim of the study and easy for the patients to answer. It evolved that the patients understood the questions very well and no changes to the guide were made. The interviews were conducted in the patients’ home or in a room in a healthcare facility by two qualified nurses from the research group. All participants were asked the same questions. The participants were interviewed two weeks after their first advance care planning conversation. This interval was chosen to enable the patients to remember the conversation and because they had possibly gained some experience of follow-up care after the conversation, while their condition had not changed greatly. In this study, advance care planning was conducted by doctors and/or by a nurse (cancer coordinator) and the patient’s family members were also invited to take part in the meeting. The nursing home ward had advance care planning guidelines based on advice from the “Centers for Medical Ethics” [35]. The content and conclusions of the advance care planning meeting were documented in a separate form in the patient record. How advance care planning is conducted, and who is responsible for conducting it, may vary in different care contexts and countries.

One limitation was the small number of participants in this study which affects the confirmability of the study. The recruitment of participants was difficult due to inadequate implementation and nurses exercising gatekeeping control. Although municipal managers and employees were positive, it turned out that the recruiters found it ethically challenging to recruit patients with a short life expectancy since they felt that many of them were too ill to participate in interviews. However, all participants invited agreed to take part in the study and spontaneously stated that they considered participation to be important and meaningful.

### Recommendations for further research

There is a need for more research on how to improve communication skills among healthcare professionals in home care to improve the quality in palliative care aiming to meet the needs of palliative patients living at home. The support and security that patients in this study experienced in nursing homes was largely absent in home care, even though the patients found the care to be good. A key aspect is that home care patients often feel lonely with great responsibility and serious illness, making them feel unsafe. There is therefore a need for more research on patients in home care in late palliative phase and their perspectives on care leading to a sense of security.

## Conclusion

Patients in late palliative phase and their experiences of palliative care highlight the need for information and support from healthcare professionals, but the organization of primary healthcare settings does not adequately address these needs. A key aspect of quality in care is comprehensive and individualized follow-up throughout the patient’s illness trajectory. Another area that affects the quality of palliative care is the communication skills of healthcare professionals. Care plans for the end- of -life were developed through advance care planning, but the quality of implementation varied in home care. A huge concern for patients in the late palliative phase was if and how their relatives received the support they needed.

## Supplementary Information

Supplementary Material 1

## Data Availability

The datasets used and/or analysed during the current study are available from the corresponding author upon reasonable request.
